# Mechanical and Biological Complications Two Years After Full-Arch Implant-Supported Prosthetic Rehabilitation: A Retrospective Clinical Study

**DOI:** 10.3390/clinpract15070134

**Published:** 2025-07-18

**Authors:** Denisa Tabita Sabău, Petra Saitos, Rahela Tabita Moca, Raluca Iulia Juncar, Mihai Juncar

**Affiliations:** 1 Doctoral School of Biomedical Sciences, University of Oradea, 1 Universității Street, 410087 Oradea, Romania; denisa.sabau@gmail.com (D.T.S.); petrasaitos@yahoo.com (P.S.); 2 Department of Dentistry, Faculty of Medicine and Pharmacy, University of Oradea, 10 Piața 1 Decembrie Street, 410073 Oradea, Romania; ralucajuncar@yahoo.ro (R.I.J.); mihaijuncar@gmail.com (M.J.)

**Keywords:** full-arch implant prostheses, edentulous patients, mechanical complications, biological complications, risk factors

## Abstract

**Background/Objectives:** Full-arch implant-supported prostheses have become a widely accepted solution for edentulous patients, yet long-term biological and mechanical complications remain a clinical concern. **Methods**: This retrospective study included 70 fully edentulous patients (362 implants) rehabilitated with either fixed or removable implant-supported prostheses. Data were collected on demographics, medical status, type and location of prostheses, implant type, abutments, method of fixation, and complications. Statistical analysis included Fisher’s exact test, the Mann–Whitney U test, and chi-squared tests, with a significance level set at *p* < 0.05. **Results**: Mechanical complications occurred in 41.4% of patients (29 out of 70), with framework fractures reported in eight cases (27.6%), ceramic chipping in six cases (20.7%), and resin discoloration in four cases (13.8%). The prostheses were fabricated using monolithic zirconia, metal–ceramic crowns, zirconia on titanium bars, and hybrid resin/PMMA on cobalt–chromium frameworks. Gingival inflammation was also noted in 41.4% of cases (*n* = 29), predominantly in posterior implant regions. Younger patients and those without systemic diseases showed a significantly higher incidence of mechanical complications. **Conclusions**: Two years post-treatment, mechanical and biological complications appear to be independent phenomena, not significantly associated with most prosthetic variables. Patient-specific factors, particularly age and general health status, may have greater predictive value than prosthetic design. Limitations of the study include its retrospective design and the lack of radiographic data to assess peri-implant bone changes.

## 1. Introduction

Total edentulism is a significant oral health problem defined by the complete absence of all teeth on one or both dental arches [1]. This condition has profound implications for fundamental functions such as mastication, speech, and facial esthetics. Additionally, it can also have significant psychological ramifications for the patient [2]. The prevalence of complete edentulism varies across countries and regions [3]. Globally, there has been an increase over the past 30 years in the prevalence and incidence of total edentulism cases; however, no significant changes have been observed across different age groups [4]. In Romania, the prevalence of total edentulism is high, especially among the elderly and those with low socioeconomic status. This phenomenon reflects a clinical reality that dentists frequently encounter [5].

Full-arch implant-supported prosthetic rehabilitation is a widely accepted and complex solution for restoring function and esthetics in completely edentulous patients, both in the maxilla and mandible [6]. In addition to improving oral health, this treatment significantly contributes to the patient’s satisfaction with mastication and esthetics, thus enhancing quality of life [7,8]. Despite the notable advancements in implant materials and digital workflows, the therapy remains prone to mechanical and biological complications, which may negatively affect long-term clinical outcomes and patient satisfaction [9].

Mechanical complications, such as framework fractures, ceramic chipping, and resin discoloration, have been frequently reported in the literature [10,11]. These failures are often associated with prosthetic design limitations, material fatigue, and excessive occlusal forces. Biological complications, such as gingival inflammation, peri-implant mucositis, and peri-implantitis, can also occur and are influenced by various patient- and implant-related factors, including hygiene maintenance, prosthetic emergence profile, and implant positioning [12,13].

Clinically, peri-implant health is defined by the absence of inflammation in the surrounding soft tissues, as evidenced by the lack of redness, swelling, and bleeding on probing [14]. The maintenance of peri-implant health is a fundamental element in long-term implant success and requires ongoing follow-up protocols, particularly in general dental practice settings [15].

Systemic health and demographic variables may further modulate complication risks. For example, patients with comorbidities such as cardiovascular disease or diabetes may have increased susceptibility to biological complications, while younger, systemically healthy patients may exert greater masticatory forces, contributing to mechanical wear and fatigue [16,17].

Given these multifactorial influences, the identification of correlations between complications and clinical or demographic parameters becomes essential for improving long-term treatment success. Numerous clinical studies and systematic reviews have extensively investigated full-arch implant-supported rehabilitations, evaluating outcomes such as prosthetic survival rates, mechanical and biological complications, and patient satisfaction [18,19]. However, relatively few of these studies have specifically focused on patient-centered characteristics, such as age, systemic health, or hygiene compliance, as well as their potential association with the incidence of mechanical and biological complications. Moreover, while both fixed and removable full-arch implant-supported prostheses have been thoroughly evaluated independently, comparative clinical data assessing these two modalities within the same patient cohort under uniform clinical protocols and follow-up criteria remain scarce in the literature [16,17,20]. The novelty of the present study lies in its integrated analysis of both mechanical and biological complications within a single, uniformly treated cohort, comparing fixed and removable full-arch prostheses and stratifying outcomes by demographic and health-related variables.

The present study aims to evaluate the incidence of mechanical and biological complications in edentulous patients treated with full-arch implant-supported prostheses over a two-year period. A two-year follow-up period was chosen for this evaluation because it represents a clinically significant timeframe during which most biological and mechanical complications tend to emerge. Previous studies have reported high prosthetic survival rates within the first 24 months [21] while also noting that the majority of early failures and complications, particularly in the maxilla, occur during this interval [22,23]. Additionally, the study explores potential associations between these complications and patient-specific or treatment-related variables, with the ultimate goal of improving individualized treatment planning and post-treatment monitoring.

## 2. Materials and Methods

### 2.1. Ethical Considerations

Ethical approval for the study was obtained by the Ethics Committee of the University of Oradea (IRB no. CEFMF/1, dated 13 April 2023). Although the study was conducted in a private dental clinic, all medical personnel involved in the treatment of patients and data collection were university-affiliated staff. A formal agreement between the clinic and the University of Oradea permits university staff to carry out clinical teaching and research activities within the clinic’s facilities. Therefore, ethical oversight and approval were provided by the University’s Ethics Committee in accordance with institutional regulations. The study was conducted in accordance with the principles outlined in the 2008 Declaration of Helsinki and its subsequent amendments.

### 2.2. Participants and Data Collection

This was a retrospective observational study based on the analysis of clinical records and follow-up data collected from a private dental clinic. The study sample consisted of 70 completely edentulous patients of both genders, presenting with either maxillary, mandibular, or bimaxillary edentulism. All patients were selected from the clinical records of a private dental clinic in Oradea, Romania. All treatments were performed between January 2020 and January 2022. A total of 362 dental implants were placed in this patient cohort. All implants used in this study were commercially available from Implant Swiss (Novodent SA, Yverdon-les-Bains, Switzerland). These implants featured an internal tapered connection and platform switching, and their surfaces were treated via double-acid etching to promote optimal osseointegration. The implants used had diameters ranging from 3.3 mm to 4.8 mm and lengths between 8 mm and 14 mm, selected based on bone availability and anatomical constraints. A standardized healing period of three months was respected before prosthetic loading in all cases. The surgical placement of dental implants was performed by one experienced oral surgeon (M.J.). After the three-month standard osseointegration period, the prosthetic rehabilitations were performed by two prosthodontic specialists (R.I.J. and D.T.S.) and included both fixed and removable full-arch prostheses. All patients were followed up for a minimum period of two years from the date of prosthesis delivery.

The choice of prosthetic materials was based on patient-specific clinical needs, occlusal requirements, esthetic demands, and clinician preference. Monolithic zirconia (Dentsply Sirona, Charlotte, NC, USA) was used for its high strength and esthetic properties, particularly in cases with adequate vertical space and high functional demands. Hybrid prostheses were fabricated using PMMA (VITA, VITA Zahnfabrik, Bad Säckingen, Germany) on cobalt–chromium frameworks, offering a lightweight and cost-effective solution, especially in patients requiring removable options or with limited restorative space. Metal–ceramic crowns and zirconia crowns on titanium bars were also selected depending on case-specific prosthetic constraints. The cobalt–chromium alloy was supplied by dental laboratories, though brand names were not consistently recorded.

A structured database was created for data collection, into which all relevant clinical and demographic parameters considered for inclusion in the study were systematically recorded.

Exclusion criteria comprised patients who failed to attend regular check-ups every six months, those receiving implants or prosthetic reconstructions from other dental clinics, and those lacking crucial data in their medical records concerning prosthetic works or implants. Patients with missing data necessary for the study’s development were also excluded.

The clinical and demographic parameters considered for inclusion in the study were as follows: gender (male, female), age, presence of systemic comorbidities, the rehabilitated arch (maxilla or mandible), the number of implants and their anatomical position (anterior or posterior regions), the implant type (standard, mini/narrow, or custom-made), the type of prosthetic abutments used (standard or multi-unit abutments [MUA]), the design of the prosthetic restoration (fixed or removable), and the retention method (cement-retained, screw-retained, or overdenture with various attachment systems).

Both mechanical and biological complications were assessed during the follow-up period. Mechanical complications included prosthetic fractures, ceramic chipping, and material degradation. Biological complications were defined based on the clinical presence or absence of gingival inflammation, as recorded by clinicians during routine follow-up visits and documented in patient files. It is important to note that the study did not distinguish between peri-implant mucositis and peri-implantitis; no radiographic evaluation of peri-implant bone levels was performed. The biological complication was thus limited to the presence of clinically visible gingival inflammation.

### 2.3. Statistical Analysis

Statistical analyses were conducted using IBM SPSS Statistics 25 and Microsoft Office Excel/Word 2013. Quantitative variables were assessed for distribution using the Shapiro–Wilk test and were presented as means with standard deviations or medians with interpercentile ranges. Non-parametrically distributed quantitative independent variables were analyzed using the Mann–Whitney U/Kruskal–Wallis H test, supplemented by Dunn–Bonferroni post hoc tests. The Friedman test, accompanied by post hoc Dunn–Bonferroni tests, was employed for non-parametrically distributed quantitative variables with repeated measures. Repeated measures ANOVA, augmented by Bonferroni post hoc tests, was used for quantitatively repeated measures with normal distribution. Statistical significance was set at a *p*-value of <0.05.

## 3. Results

### 3.1. Sample Characteristics

The study included a total of 70 completely edentulous patients, rehabilitated at either the maxillary, mandibular, or both arches. Across this cohort, a total of 362 dental implants were placed. All patients were monitored for a period of two years following prosthetic loading, with routine clinical evaluations performed every six months.

Out of the 70 cases, the majority were male patients (57.1%, *n* = 40), with a mean age of 57.4 years (SD ± 9.41) and a median age of 60 years (range: 37–73). Half of the patients (50%, *n* = 35) presented with systemic comorbidities. The most frequently reported were cardiovascular disorders, including hypertension and arrhythmias (22 cases; 62.85%), followed by endocrine conditions such as diabetes mellitus (14 cases; 40%). Less common comorbidities included hepatic disorders (two cases; 5.71%), psychiatric conditions (one case; 2.85%), hematological disease (leukemia; one case; 2.85%), and neurological disorders (poliomyelitis; one case; 2.85%).

Rehabilitations were predominantly performed in the maxilla (62.9%, *n* = 44). The most common number of implants used per arch was either four (41.4%) or six (42.9%). Implant distribution patterns most often included two anterior and two posterior implants (51.4%, *n* = 36). Standard implants were used in most cases (91.4%, *n* = 64), and multi-unit abutments (MUAs) were employed in 67.1% (*n* = 47) of restorations.

Fixed prosthetic restorations were more frequently used than removable ones, with 72.9% (*n* = 51) of cases involving fixed restorations. Among these, a monolithic zirconia structure was used in 25.5% of cases (*n* = 13), metal–ceramic crowns in 27.5% (*n* = 14), zirconia crowns on titanium bars in 13.7% (*n* = 7), and hybrid resin/PMMA on cobalt–chromium bars in 33.3% cases (*n* = 17). Removable prostheses were used in 27.1% of cases (*n* = 19), of which 68.4% were retained with bar attachments (*n* = 13) and 31.6% with locator systems (*n* = 6).

In terms of retention, screw-retained prostheses were the most frequently used (38.2%, *n* = 26), followed by cemented restorations (29.4%, *n* = 20).

No definitive failures were recorded during the two-year follow-up. None of the implants were lost, and no prostheses required full replacement. All mechanical complications were classified as technical in nature and were managed conservatively through maintenance or minor repairs. Specifically, fractures, ceramic chipping, and resin discoloration did not necessitate prosthesis remaking and were resolved through chairside adjustments or laboratory repair procedures.

### 3.2. Prevalence and Type of Complications

At the two-year follow-up, mechanical complications were observed in 41.4% of patients (*n* = 29). The most frequent mechanical issues included framework fractures (27.6%, *n* = 8), ceramic chipping (20.7%, *n* = 6), and resin discoloration (13.8%, *n* = 4). Fractures were primarily located in the connector zones of monolithic zirconia restorations, whereas chipping predominantly occurred in metal–ceramic prostheses. Discoloration was mainly noted in hybrid resin restorations.

Biological complications, defined in this study as clinically evident gingival inflammation (in the absence of a radiographic evaluation for bone loss), were also present in 41.4% of the patient cohort (*n* = 29). Inflammation was most commonly observed in posterior implant regions (93.1%, *n* = 27) and was noted in both anterior and posterior sites in more than half of the affected cases (55.5%, *n* = 15) (Table 1).

Notably, there was no statistically significant association between the occurrence of mechanical and biological complications (*p* = 0.460), suggesting that these two types of complications may arise independently during the early post-prosthetic period.

### 3.3. Statistically Significant Associations

**Figure 1:**
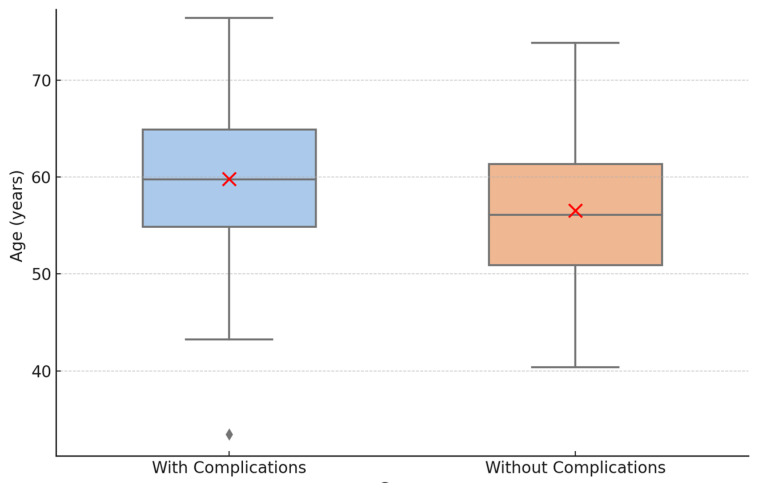
Age distribution by mechanical complications status. ♦ Represents an outlier, a value that lies outside the typical range of the data.

A statistically significant difference was found between age and the presence of mechanical complications. Patients who experienced mechanical complications had a significantly lower median age (58 years; IQR: 44–64) compared to those without complications (61 years; IQR: 51–66; *p* = 0.037, Mann–Whitney U test). This finding may suggest a potential relationship between younger age and increased mechanical stress or usage, possibly due to greater occlusal forces and higher levels of physical activity (Table 2; Figure 1).

**Figure 2:**
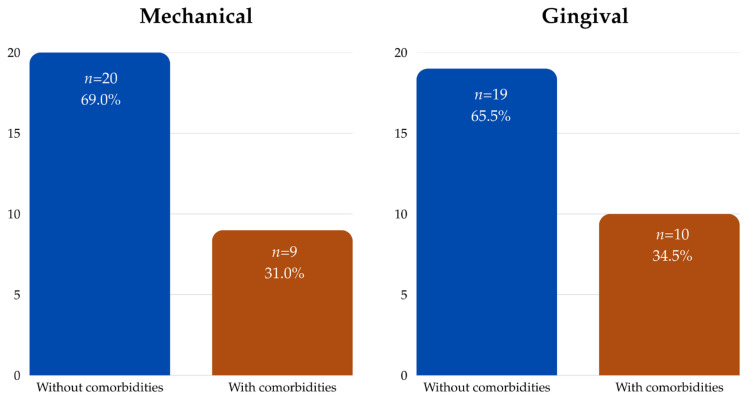
Complications rates by comorbidity status.

Additionally, systemic health status emerged as a significant variable. Patients without systemic comorbidities demonstrated a higher incidence of both mechanical (69.0% vs. 36.6%; *p* = 0.015) and biological complications (65.5% vs. 39.0%; *p* = 0.029) compared to those with comorbidities (Table 3; Figure 2). These results are counterintuitive and suggest that healthier individuals might place greater functional demands on their prostheses or possibly have lower adherence to maintenance recommendations.

### 3.4. Non-Significant Associations

No statistically significant associations were found between the occurrence of complications (mechanical or biological) and several clinical variables, including arch location (maxilla vs. mandible) (*p* = 1.000), the number of implants per arch (*p* = 0.712), implant position (anterior vs. posterior) (*p* = 0.550), the type of implant (standard, mini/narrow, or custom) (*p* = 0.339), the type of prosthetic abutment (standard vs. MUA) (*p* = 0.131), the type of prosthetic restoration (fixed vs. removable) (*p* = 0.283), and the retention method (screw, cement, or overdenture) (0.508).

These findings suggest that patient-related factors, particularly age and general health status, may be more influential in the development of complications than the technical or design-related aspects of the prosthetic intervention.

## 4. Discussion

This study aimed to investigate the incidence of mechanical and biological complications following full-arch implant-supported rehabilitations and to explore their potential associations with patient-related and treatment-related variables. One of the most notable and unexpected findings was the significantly higher complication rate observed in younger and systemically healthy patients. This challenges common assumptions that older age or comorbidities are the main predictors of prosthetic failure and suggests that behavioral or biomechanical factors may play a more decisive role in early post-treatment outcomes.

Mechanical complications documented at the two-year follow-up exceeded expectations, affecting 41.4% (*n* = 29) of patients. These included fractures in monolithic zirconia structures, typically at the connector area between two implants, and ceramic chipping in metal-ceramic restorations. Discoloration issues were also reported in hybrid resin-based rehabilitations. The current literature confirms these types of complications [24,25]. In a comprehensive evaluation, Sailer I. et al. investigated the survival rates and complication profiles of implant-supported metal–ceramic and zirconia–ceramic restorations. The findings revealed that metal–ceramic restorations exhibited a 5-year fracture and chipping rate of 11.6%, whereas zirconia-based implant prostheses showed a significantly higher complication rate of 50% (*p* < 0.001) [26]. Other studies have highlighted the occurrence of mechanical complications in metal–ceramic structures, such as fractures and ceramic chipping [27,28].

Other studies have assessed the long-term performance of full-arch fixed implant-supported prostheses fabricated from monolithic zirconia. The researchers observed that fractures occurring in the connector regions between implants represent a significant clinical concern, emphasizing the critical importance of appropriate connector design to prevent such complications [29,30,31].

Discoloration was reported in 13.8% of patients (*n* = 4) and was mainly associated with hybrid resin-based prosthetic restorations. Most affected cases involved PMMA composite materials veneered on cobalt–chromium bars. These esthetic complications may reflect the inherent limitations of PMMA, such as increased water absorption, surface porosity, and low resistance to pigment infiltration, particularly in patients with a high consumption of staining agents or suboptimal oral hygiene. Over time, these factors contribute to a progressive loss of color stability, impacting the esthetic longevity of the prostheses. These observations are consistent with the literature, which consistently reports higher discoloration rates in resin-based materials compared to ceramic alternatives [32,33,34].

Although our study did not reveal significant differences in the incidence of mechanical complications based on patient sex, age-related trends were evident. Patients who developed mechanical complications within the two-year follow-up period were significantly younger than those who did not experience such issues. This observation is consistent with the existing literature, which identifies age as a relevant factor in the incidence of mechanical failures in implant-supported restorations. In the past five years, several studies have investigated the relationship between patient age and the occurrence of mechanical complications in implant-supported prostheses. A recent study published assessed risk factors associated with dental implant failure and found that younger patients are at a higher risk of experiencing mechanical complications compared to older individuals. The authors suggest that this may be due to stronger masticatory forces and higher levels of physical activity in younger populations [35]. A similar finding was highlighted in another study from 2020, which analyzed the incidence of mechanical complications in implant-supported fixed prostheses over a two-year period. The study also reported that patients who experienced mechanical complications were, on average, younger than those without such issues [36].

Another notable observation was that patients without systemic comorbidities had a significantly higher incidence of mechanical complications at two years (69%) compared to those with comorbidities (36.6%). This may suggest that systemic health conditions do not necessarily correlate with a higher risk of mechanical failure, a conclusion supported by other recent studies. Similarly, studies have shown that the relationship between comorbidities and mechanical complications in patients with dental implants have reported comparable findings. A study published last year assessed risk factors for mechanical complications in implant-supported restorations and found that patients without systemic diseases exhibited a higher incidence of complications two years after treatment compared to those with underlying health conditions. The authors attributed this outcome to the possibility that healthier individuals tend to have more intense physical activity and more frequent use of their prostheses, leading to increased mechanical wear [37].

Younger patients were found to be more prone to mechanical complications compared to older patients, a result corroborated by recent studies indicating increased masticatory forces and functional activity in younger individuals.

Occlusal factors are closely associated with mechanical implant complications, while their influence on biological outcomes remains unclear. Although not directly implicated in peri-implant disease, occlusal overload may worsen inflammatory conditions. Understanding biomechanics is key to minimizing risks [38].

Contrary to certain expectations, an important finding of this study is the absence of statistically significant associations between the occurrence of mechanical complications and the other parameters evaluated, namely the number and type of implants, their arch location, the type of prosthetic abutments, the type of prosthetic restoration (fixed or removable), and the retention method. This may suggest that these variables do not exert a major influence on the incidence of complications, or alternatively, that the sample size was insufficient to detect potential differences.

Biological complications were classified as the presence of gingival inflammation, identified clinically through visual inspection alone without peri-implant probing. This approach was adopted to minimize the risk of iatrogenic trauma to the peri-implant tissues and to ensure consistency in diagnostic assessment, particularly during the early post-rehabilitation period.

Peri-implant health is clinically characterized by the lack of inflammatory signs, as determined through visual inspection—specifically, the presence of pink (rather than erythematous) mucosa, the absence of swelling, and a firm consistency of the surrounding soft tissues [39]. The clinical evaluation of peri-implant soft tissue health should involve an assessment of overall oral hygiene, with particular attention to the presence of biofilm on implant surfaces and their restorative components.

Dental implants should undergo routine visual inspection and periodontal probing at regular intervals—ideally at least once per year—as part of comprehensive oral examinations, following a protocol comparable to that used for natural dentition [40,41].

The biological complications of dental implants primarily manifest as inflammation of the peri-implant soft and hard tissues, often involving the prosthetic components, and they are mainly driven by bacterial biofilm accumulation. Clinically, these conditions are categorized as peri-implant mucositis or peri-implantitis [40,42,43].

Recent evidence suggests that the peri-implant mucosal seal exhibits greater susceptibility to bleeding on probing (BoP) than gingival tissues surrounding natural teeth, even in clinically healthy sites. Recent publications showed that a greater physiological probing depth, with a higher average of bleeding on probing, should be expected around implants compared to teeth, even in a healthy condition [44]. Similarly, a 2021 article in the Journal of Periodontology highlights that bleeding on probing (BoP) around implants may result from either traumatic or pathological probing, introducing diagnostic ambiguity and complicating the clinical interpretation of peri-implant tissue status [45].

Bleeding on probing around implants may arise not only from excessive probing force but also from difficulties in accessing the peri-implant sulcus, often due to prosthetic contours and implant–restoration positioning. Moreover, the absence of a periodontal ligament and variations in prosthetic design further compromise the reliability and interpretation of probing depth measurements in peri-implant assessments [46].

Gingival inflammation was reported in 41.4% of patients at the two-year follow-up, with 93.1% of these cases involving posterior implants. Patients without systemic conditions had a higher incidence of gingival inflammation. No significant correlations were found between inflammation and patient sex, age, implant type, or attachment method. This relatively high rate of soft tissue inflammation may be explained by a combination of patient- and treatment-related factors. Although oral hygiene levels and prosthetic emergence profiles were not directly analyzed in this study, it is known that posterior implant sites present greater challenges for plaque control [47]. Furthermore, the higher inflammation rate observed in younger, systemically healthy patients may reflect lower compliance with maintenance protocols or reduced awareness of peri-implant care requirements [48]. The literature also reports higher inflammation rates in the posterior regions of full-arch rehabilitations, attributed to the challenges of hygiene access [49].

The relationship between mechanical complications and gingival inflammation was also analyzed. Both were observed in 41.4% of the studied cases at the two-year follow-up after implant–prosthetic rehabilitation. However, no significant association was found between the presence of mechanical complications and gingival inflammation at this time point. This finding is supported by the existing literature, which indicates that while mechanical complications and gingival inflammation may occur concurrently, there is no direct correlation between the two within this time frame. A 2018 systematic review assessing mechanical and biological complications of full-arch implant-supported restorations over a two-year period similarly concluded that the incidence of mechanical complications, such as fractures or screw loosening, was not significantly correlated with the presence of gingival inflammation or peri-implantitis during that period [50].

While complications like mucositis and peri-implantitis are more common in full-arch rehabilitations, they are often linked more to post-operative hygiene than to the type of prosthetic design [51,52].

Early implant failure has been shown to vary by anatomical site, with higher rejection rates reported in the buccal and lingual mandibular regions during the first months post-insertion, while gender appeared to have no impact on outcomes [53]. These findings highlight the importance of anatomical and biomechanical factors, which may also influence the incidence of complications in full-arch prosthetic rehabilitations.

The rehabilitation of edentulous patients using fixed, removable, or combined prosthodontic approaches has proven effective over the medium to long term, particularly when guided by individualized treatment planning, which enhances both functional and esthetic outcomes, as well as quality of life [54].

Our research contributes to the literature by confirming that the number and type of implants do not significantly influence gingival inflammation, suggesting that long-term success is more dependent on maintenance and hygiene protocols.

Despite the occurrence of mechanical complications and gingival inflammation, these outcomes do not appear to be significantly influenced by treatment-related parameters. This suggests that the long-term success of prosthetic rehabilitations may depend more on factors not assessed in the present study, such as patient-specific biomechanics, behavioral patterns, and adherence to post-treatment care protocols. Similar findings have been reported in studies analyzing maxillofacial trauma, where soft tissue damage and fracture complexity were more strongly associated with the kinetic energy of the trauma and patient-related variables rather than the anatomical fracture site itself [55].

This study has several limitations that should be acknowledged. First, the relatively small sample size (70 patients), the single-center design, and the two-year follow-up period may limit the extent to which the findings reflect long-term clinical outcomes or can be applied across broader populations. Future studies should aim for larger, multicenter cohorts with extended observation periods to improve the applicability and depth of the findings.

Second, the assessment of complications was based exclusively on clinical evaluations, without standardized radiographic monitoring of peri-implant bone levels. This limitation may have led to an underestimation of peri-implantitis and hindered the ability to distinguish it from peri-implant mucositis. Incorporating regular radiographic assessment would enhance diagnostic accuracy and the early detection of biologic complications.

Third, the condition of the opposing dentition was not systematically recorded. Factors such as the presence of natural teeth, ceramic restorations, or removable prostheses in the antagonistic arch, as well as oral hygiene status, could influence both occlusal forces and plaque accumulation. These unmeasured variables may have contributed to mechanical or biological complications and should be considered in future investigations.

Finally, the relatively limited diversity of systemic comorbidities in the study population may restrict insights into how the general health status interacts with implant performance. Despite these limitations, the present study offers valuable information on the complication profiles associated with full-arch implant-supported prostheses and highlights the need for future research into material-specific complication rates, maintenance strategies, and long-term success predictors.

## 5. Conclusions

This study highlights a clinically relevant rate of complications at two years post-rehabilitation, with mechanical failures and gingival inflammation occurring independently of the assessed technical and demographic variables. Younger, systemically healthy patients showed higher complication rates, possibly due to greater functional demands or lower compliance with maintenance protocols.

Material-specific trends were observed: zirconia restorations were prone to framework fractures, metal-ceramic prostheses to chipping, and hybrid resin/PMMA to discoloration. These findings emphasize the need for personalized maintenance and careful material selection to optimize both functional and esthetic outcomes.

## Figures and Tables

**Table 1:** Distribution according to different variables.

Parameter/Category	Details/Distribution
Gender	Male: 40 (57.1%); female: 30 (42.9%)
Age	Mean ± SD: 57.4 ± 9.41 Median: 60
Type of Comorbidity	Cardiovascular: 22 (62.85%) Endocrine: 14 (40%) Hepatic disorders: 2 (5.71%) Psychiatric conditions: 1 (2.85%) Hematologic: 1 (2.85%) Neurological: 1 (2.85%)
Implant Location	Maxillary: (62.9%) Mandibular: (37.1%)
Number of Implants per Arch	Four implants: 29 (41.4%) Six implants: 30 (42.9%) Other configurations: 11 (15.7%)
Types of Implants Used	Standard implants: 64 (91.4%) Other types: 6 (8.6%)
Type of Restorations	Fixed: 51 (72.9%) Removable: 19 (27.1%)
Materials Used for Fixed Restorations	Monolithic zirconia: 13 (25.5%) Metal–ceramic crowns: 14 (27.5%) Zirconia crowns on titanium bars: 7 (13.7%) Hybrid resin/PMMA on Co-Cr bars: 17 (33.3%)
Attachment Systems for Removable Prostheses	Bar attachments: 13 (68.4%) Locator systems: 6 (31.6%)
Mechanical and Biological Complications (2-Year Follow-Up)	Present: 29 (41.4%) Absent: 41 (58.6%)
Mechanical Complications	Framework fractures: 8 (27.6%) Ceramic chipping: 6 (20.7%) Resin discoloration: 4 (13.8%)

**Table 2:** Age distribution by mechanical complication status.

Mechanical Complications	Mean ± SD	Median (IQR)	*p*-Value
Present (*n* = 29)	56.2 ± 8.9	58 (44–64)	0.037 *
Absent (*n* = 41)	59.6 ± 9.7	61 (51–66)	

* Mann-Whitney U test.

**Table 3:** Complication rates by comorbidity status.

Comorbidities	Mechanical Complications				*p* *
	Absent		Present		
	No.	%	No.	%	
No Comorbidities	15	36.6%	20	69%	0.015
With Comorbidities	26	63.4%	9	31%	
	**Gingival Complications**				*p* *
	**Absent**		**Present**		
	**No.**	**%**	**No.**	**%**	
No Comorbidities	16	39%	19	65.5%	0.029 *
With Comorbidities	25	61%	10	34.5%	

* Pearson chi-squared test.

## Data Availability

The data presented in this study are available upon request from the corresponding author due to privacy and ethical restrictions related to patient confidentiality.
